# Fast-Track Ultrasound Clinic for the Diagnosis of Giant Cell Arteritis Changes the Prognosis of the Disease but Not the Risk of Future Relapse

**DOI:** 10.3389/fmed.2020.589794

**Published:** 2020-12-08

**Authors:** Sara Monti, Alice Bartoletti, Elisa Bellis, Paolo Delvino, Carlomaurizio Montecucco

**Affiliations:** ^1^Rheumatology Department, IRCCS Policlinico S. Matteo Foundation, University of Pavia, Pavia, Italy; ^2^PhD in Experimental Medicine, University of Pavia, Pavia, Italy

**Keywords:** giant cell arteritis, sonography, ultrasoud, fast-track, permanent visual loss, large vessel vasculitis

## Abstract

**Background:** Color Duplex sonography (CDS) of temporal arteries and large vessels (LV) is a recently validated diagnostic methodology for Giant Cell Arteritis (GCA). CDS combined with a fast-track approach (FTA) has improved the early diagnosis of the disease.

**Objectives:** To assess FTA effects on the prevention of permanent visual loss (PVL), relapse and late complications of GCA compared to conventional practice. To assess the impact of COVID-19 pandemic on outcomes of GCA patients assessed with FTA.

**Methods:** GCA patients diagnosed up to June 2020 at the Rheumatology Department, University of Pavia, were included. FTA was implemented since October 2016. FTA consists in the referral within 1 working day of a suspected GCA case to an expert rheumatologist who performs clinical evaluation and CDS.

**Results:** One hundred sixty patients were recruited [female 120 (75%), mean age 72.4 ± 8.2 years]. Sixty-three (39.4%) evaluated with FTA, 97 (60.6%) with conventional approach. FTA patients were older (75.1 ± 7.6 vs. 70.6 ± 8.2 years old; *p* < 0.001). Median follow-up duration was shorter in the FTA group compared to the conventional one (0.9 vs. 5.0 years; *p* < 0.001). There was no difference between the two cohorts regarding major vessel district involvement (LV-GCA 17.5% vs. 22.7%; *p* = 0.4). PVL occurred in 8 (12.7%) FTA patients and 26 (26.8%) conventional ones (*p* = 0.03). The relative risk of blindness in the conventional group was 2.11 (95% C.I. 1.02–4.36; *P* = 0.04) as compared to FTA. Median symptom latency of patients experiencing PVL was higher in the conventional group (23 days IQR 12–96 vs. 7 days IQR 4–10, *p* = 0.02). During COVID-19 there was a significant increase in the occurrence of PVL (40%) including bilateral blindness despite a regularly operating FTA clinic. Cumulative incidence of relapses and time to first relapse did not change after FTA introduction (*P* = 0.2). No difference in late complications (stenosis/aneurysms) was detected.

**Conclusions:** FTA including CDS evaluation contributed to a substantial reduction of PVL in GCA by shortening the time to diagnosis and treatment initiation. Relapse rate did not change upon FTA introduction, highlighting the need for better disease activity monitoring and treatment strategies optimization based on risk stratification that would predict the occurrence of relapse during glucocorticoid de-escalation.

## Introduction

Giant Cell Arteritis (GCA) is the most prevalent primary systemic vasculitis (1). GCA typically affects the aorta and its major branches, even though medium and small-sized arteries may be targeted as well (2). Indeed, permanent visual loss (PVL), one of the most feared complications of GCA, results from damage to ophthalmic, retinal or ciliary arteries (3). Other clinical manifestations include headache, jaw or tongue claudication, scalp tenderness and polymyalgia rheumatica (PMR). Sometimes the picture is protean with increased inflammatory markers, fever and weight loss and it may get underdiagnosed. The heavy morbidity burden of GCA is due to its rather late onset (usually occurring over the age of 50 years old) and the occurrence of PVL in approximately 20% patients, usually at the very beginning of the disease. Treatment with high-dose glucocorticoids (GC) should be promptly initiated when the disease is suspected to prevent PVL. The clinical spectrum of GCA also comprises large vessel complications such as thoracic and abdominal aortic aneurysm or dissection, which can increase the risk of mortality (2, 4).

Strategies for early GCA diagnosis have been implemented in the last decade in order to reduce the occurrence of blindness. Color-duplex sonography (CDS) of temporal arteries (TAs) and large extra-cranial vessels (LVs) has been recently recognized as a first-line diagnostic tool for patients with suspected GCA in centers with the adequate expertise in the technique. A fast track approach (FTA), incorporating CDS has been associated with a significant reduction of PVL in two retrospective studies (5, 6). However, some patients still manifest with PVL as the very first symptom of this disease.

The role of imaging, including CDS, as a prognostic tool and in the follow-up of patients with GCA including the detection of relapses is still controversial (7).

## Objectives

The aim of this study is to assess the role of FTA on PVL prevention and long-term relapse risk and outcome of patients with GCA, comparing the FTA outcomes with conventional practice while taking into-account the influence of the severe acute respiratory syndrome coronavirus-2 (SARS-CoV-2) pandemic on the FTA activity.

## Methods

The present study included patients who were diagnosed with GCA between January 1st, 2005 and June 30th, 2020 at the Rheumatology Department of IRCCS Fondazione Policlinico San Matteo hospital in Pavia, Italy.

The study has been approved by the Institutional Review Board of the University of Pavia and patients consented to their data use.

FTA was implemented since October 1st, 2016. FTA consists in the referral within one working day of a suspected GCA case to an expert rheumatologist, who performs the clinical examination and CDS of temporal and axillary arteries. Other anatomical sites (e.g., facial arteries, occipital arteries, carotid arteries) could be assessed according to the patient's clinical picture. Patients can be referred to the FTA from the general practitioner or other Departments. Patients enrolled after October 2016 were considered FTA if they received a rheumatologist's evaluation within one working day after the first medical contact. Patients assessed as part of the FTA between March 1st and June 30th 2020 during the coronavirus 2019 (COVID-19) pandemic were analyzed separately. Patients assessed prior to October 2016 were included in the conventional practice group (no-FTA).

The diagnosis of GCA was confirmed by the expert rheumatologist on the basis of typical symptoms, data on inflammatory markers, and imaging (CDS, ^18^FDG PET-CT) or temporal artery biopsy (TAB) findings. Since the FTA implementation, the same rheumatologist (S.M.) performed the clinical and ultrasonographic assessment.

Clinical manifestations at disease onset, laboratory tests, date of symptoms onset, date of diagnosis, comorbidities, information on treatment, and follow-up, including relapses were extracted from the clinical records and recorded on an electronic database. Data were extracted retrospectively for the conventional cohort. FTA patients were assessed prospectively since diagnosis. Treatment followed current recommendations for both cohorts including high-dose GC (1 mg/kg/day; maximum 60 mg/day) maintained for 1 month and then tapered while monitoring disease activity. In patients with recent onset of ischaemic visual symptoms i.v. methylprednisolone was prescribed for the first 3 days (1 g/day). Adjunctive immunosuppressive treatment (most frequently Methotrexate or Tocilizumab) were prescribed to relapsing patients (8, 9). PVL was defined by the complete or partial visual impairment caused by vasculitic damage of the visual pathway [e.g., anterior ischaemic optic neuropathy (AION); posterior ischaemic optic neuropathy (PION)] confirmed after ophthalmologic assessment. Symptom latency was calculated as the time interval between the onset of the first symptom attributable to GCA (e.g., date of headache or other cranial symptoms onset, girdle inflammatory pain onset, systemic symptoms onset) and the diagnosis. Large-vessel GCA (LV-GCA) was defined as the presence of extra-cranial LV involvement (e.g., aortic, axillary arteries) detected by imaging (most frequently CDS and/or ^18^FDG PET-CT) with or without temporal artery involvement. Relapse was defined as the reoccurrence of symptoms and signs of GCA, with or without elevation of the inflammatory markers, with the need to increase the dosage of GC or to add or increase the dose of another immunosuppressive drug. Disease complications during follow-up are defined by the occurrence of stenosis or aneurysms/dissections and/or ischaemic complications.

### Ultrasonographic Assessment

CDS was performed by the same rheumatologist experienced in vascular ultrasound for the assessment of large vessel vasculitis (S.M.). Patients were assessed with the MyLab Seven Esaote ultrasound machine with a high-frequency (18–6 MHz) linear transducer. Focus was set at 5 mm for temporal arteries and 3 cm for axillary arteries. A Doppler frequency of 10 MHz was applied. Pulse repetition frequency (PRF) was set at 2–3 KHz for temporal artery, and at 3–4 KHz for axillary arteries. A low wall filter was selected to allow the identification of low velocity flow. The color box was adjusted to obtain an angle steer correction ≤ 60 degrees.

The common, parietal, and frontal branches of each temporal artery and the axillary arteries in longitudinal and transverse plans were assessed at each visit. The presence of a halo was defined according to the accepted definitions as a homogenous, hypoechoic wall thickening, well-delineated toward the luminal side, visible both in longitudinal and transverse planes, most commonly concentric in transverse scans (10). The compression sign was applied in transverse views to confirm the findings (11). CDS was defined as positive if displaying a halo at the level of at least one branch of the temporal artery or at least one axillary artery. Intima-media thickness was measured at the site of maximum thickness on longitudinal plans.

### Statistical Analysis

Categorical variables were presented as numbers and percentages. Continuous variables were displayed as means with standard deviation (S.D.) if normally distributed or as medians with interquartile range (IQR) in case of non-normal distribution. Ratios were presented with 95% confidence intervals (C.I.). For comparison between groups, the independent-samples *t*-test for continuous variables and the chi-squared test for categorical variables were applied. For non-parametric numerical variables, the Wilcoxon signed-rank test was used. Statistical analysis was performed the software RStudio (R version 3.6.3 2020-02-29, Copyright © 2020 The R Foundation for Statistical Computing). *P* < 0.05 were considered to be significant.

## Results

### Baseline Assessment

One-hundred and sixty patients were recruited, 120 were females (75%), the mean age at diagnosis was 72.4 ± 8.2 years. Sixty-three (39.4%) were evaluated with FTA, while 97 (60.6%) underwent the conventional approach.

FTA patients were older (75.1 ± 7.6 vs. 70.6 ± 8.2 years old; *p* < 0.001). At the time of diagnosis, a larger percentage of comorbidity-free patients was observed in the non-FTA group (16.7 vs. 3.8%; *P* = 0.02). Nevertheless, major cardiovascular comorbidities like coronary artery disease, dyslipidaemia, diabetes, stroke, peripheral artery disease and hypertension were similar between the two cohorts (*P* > 0.05) except for heart failure (1.0% conventional vs. 7.5% FTA; *P* = 0.03). Overall, 19.4% (ranging 15.5–25%) of patients with newly diagnosed GCA had a previous diagnosis of polymyalgia rheumatica.

FTA contributed to shorten the time to diagnosis (62 days IQR 20–122 in the conventional cohort vs. 33 days IQR 15–83 in the FTA cohort; *P* = 0.035) (Table 1), especially for cranial GCA patients (61 days IQR 15–107 vs. 30 days IQR 13–53; *P* = 0.007). Of note, among patients with PVL the duration of symptoms (since the onset of the first symptom attributable to GCA) before receiving a diagnosis of GCA was higher in the conventional group (23 days IQR 12–96 vs. 7 days IQR 4–10; *P* = 0.02).

**Table 1:** Clinical features of the cohort at baseline.

	**Total (*N* = 160)**	**Conventional (*N* = 97)**	**FTA (*n* = 63)**	***P*-value**
Age at diagnosis, mean (S.D.), years	72.4 (8.2)	70.6 (8.2)	75.1 (7.6)	<0.001
Female, *n* (%)	120 (75)	77 (79.4)	43 (68.3)	0.1
Previous PMR, *n* (%)	31 (19.4)	15 (15.5)	16 (25.4)	0.1
Symptom latency, median (IQR), days	46 (15–101)	62 (20–122)	33 (15–83)	0.04
Symptoms latency of patients with PVL, median (IQR), days	14 (8–65)	23 (12–96)	7 (4–10)	0.02
**Comorbidities**				
Coronary heart disease	15 (10.1)	9 (9.4)	6 (11.3)	0.7
Dyslipidaemia	30 (20.1)	18 (18.8)	12 (22.6)	0.6
Diabetes mellitus	18 (12.1)	14 (14.6)	4 (7.5)	0.2
Stroke	14 (9.4)	11 (11.5)	3 (5.7)	0.2
Peripheral artery disease	7 (4.7)	5 (5.2)	2 (3.8)	0.2
Hypertension	85 (57.0)	53 (55.2)	32 (60.4)	0.5
Heart failure	5 (3.4)	1 (1.0)	4 (7.5)	0.03
Other comorbidities	102 (68.5)	73 (76.0)	39 (73.6)	0.7
No comorbidities	18 (12.1)	16 (16.7)	2 (3.8)	0.02

There was no difference between FTA and the conventional group regarding major vessel district involvement (cranial GCA 82.5 vs. 77.3%, respectively; *P* = 0.4). The clinical presentation was similar between the two groups: the frequency of PMR, headache, amaurosis fugax, jaw claudication and fever did not change between the two cohorts (*P* > 0.05). Some symptoms were more frequently recorded in the FTA group compared to the conventional practice: weight loss (30.2 vs. 13.4%; *P* = 0.01), tongue claudication (11.1 vs. 1.0%; *P* = 0.004) and scalp tenderness (20.6 vs. 5.2%; *P* = 0.003). PVL occurred in 8 (12.7%) patients in the FTA group compared to 26 (26.8%) in the conventional one (*P* = 0.03). The relative risk of blindness in the conventional group was 2.11 (95% C.I. 1.02–4.36; *P* = 0.04) as compared to FTA. The overall occurrence of ischaemic manifestations of GCA (AION, PION and cerebrovascular accidents) was higher in the non-FTA cohort (28.9 vs. 12.7; *P* = 0.02) (Table 2). The relationship between symptoms latency in the whole cohort and visual outcomes, and visual survival over time are shown in Supplementary Figures 1, 2.

**Table 2:** Clinical picture at the time of diagnosis in the two cohorts: conventional approach (No FTA) vs. FTA.

	**No FTA (*N* = 97)**	**FTA (*N* = 63)**	***P*-value**
**Symptoms at diagnosis**			
Constitutional (fever or weight loss), *n* (%)	36 (37.1)	28 (44.4)	0.4
Fever ≥ 38°C (≥100.4 F), *n* (%)	32 (33.0)	17 (27.0)	0.4
Weight loss ≥2 kg, *n* (%)	13 (13.4)	19 (30.2)	0.01
Any cranial symptom, *n* (%)	88 (90.7)	53 (84.1)	0.2
Any ocular symptom, *n* (%)	37 (38.1)	17 (27.0)	0.1
Permanent visual loss, *n* (%)	26 (26.8)	8 (12.7)	0.03
Amaurosis fugax, *n* (%)	14 (14.4)	7 (11.1)	0.5
Diplopia, *n* (%)	4 (4.1)	1 (1.6)	0.4
Jaw claudication, *n* (%)	33 (34.0)	29 (46.0)	0.1
Tongue claudication, *n* (%)	1 (1.0)	7 (11.1)	0.004
Scalp tenderness, *n* (%)	5 (5.2)	13 (20.6)	0.003
Headache, *n* (%)	77 (79.4)	45 (71.4)	0.3
PMR, *n* (%)	48 (49.5)	32 (50.7)	0.9
Increased ESR/CRP, *n* (%)	79 (90.8)	59 (96.7)	0.2
ESR (mm/h), median (IQR)	82 (48–102)	78 (63–96)	0.5
CRP (mg/L), median (IQR)	35 (20–99)	59 (24–98)	0.5
Anemia (<12 g/dL in females, <13 g/dL in males), *n* (%)	38 (65.5)	17 (77.2)	0.3
Hemoglobin, mean (S.D.), g/dL	11.4 (1.6)	11.5 (1.4)	1.0
Cranial GCA	75 (77.3)	52 (82.5)	0.4
LV-GCA	22 (22.7)	11 (17.5)	0.4
Ischaemic GCA	28 (28.9)	8 (12.7)	0.02

Forty-four (45.4%) patients of the conventional cohort underwent TAB, with 25 (56.8%) samples were compatible with GCA at the histological examination. TAB was performed in 3 patients of the new cohort, two of them without signs of active vasculitis and one with a sampling error. TAB sensitivity was 53.2% (95% C.I. 38.1–67.9%).

**Figure 1:**
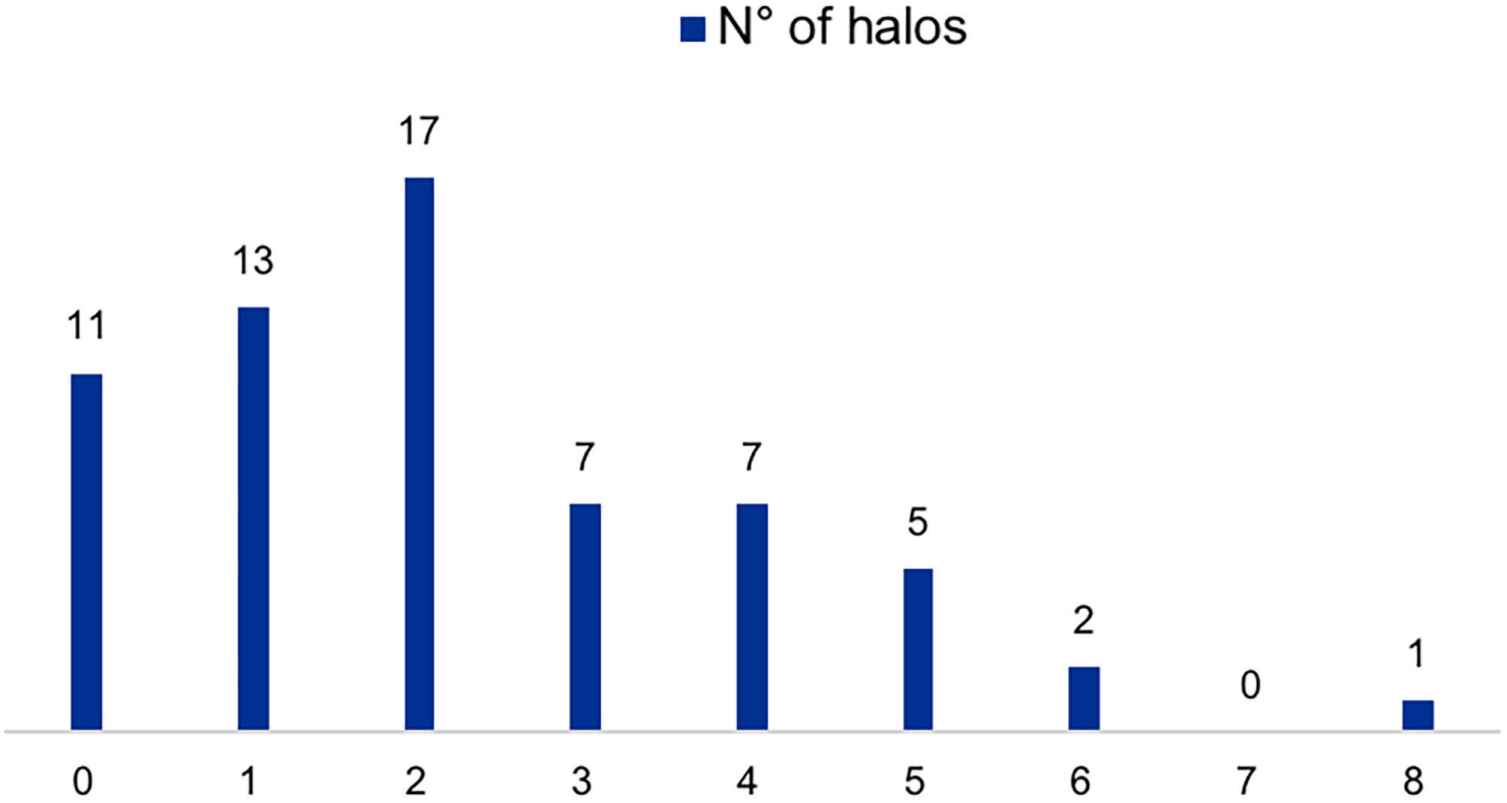
CDS findings of patients evaluated with the FTA. Number of patients according to number of sites with halos (0–8 sites including the different branches of the temporal artery and the axillary arteries).

**Figure 2:**
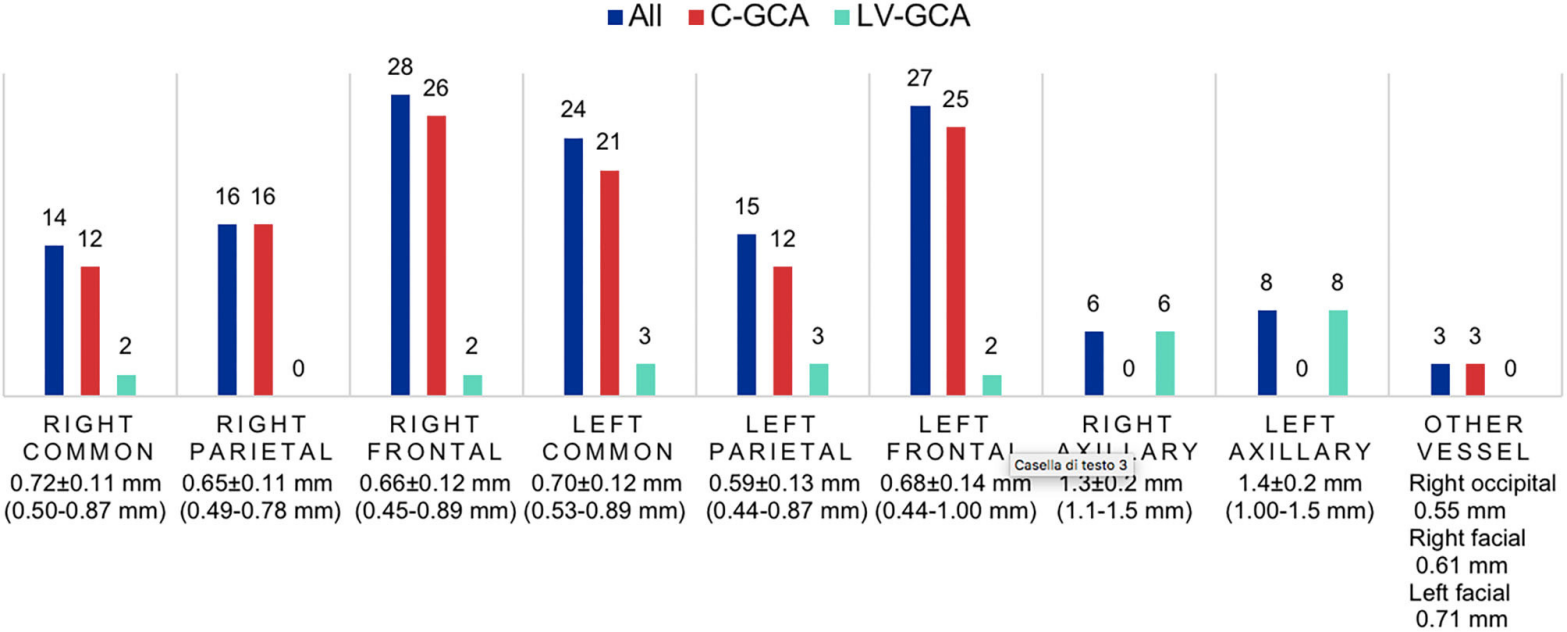
CDS findings distribution and intima-media thickness of patients evaluated with the FTA. Number of patients with positive CDS in each vascular district, the average halo thickness (± S.D., range) is reported below. C-GCA, cranial giant cell arteritis; LV-GCA, large-vessel giant cell arteritis.

Within the FTA group, all 63 (100.0%) were evaluated with CDS at the time of diagnosis, 52 of them having specific ultrasound findings of GCA (Figures 1, 2), yielding a sensitivity of 82.5% (95% C.I. 70.9–91.0%). Thirty-one (49.2%) had bilateral halo at CDS. Forty-three presented with halo sign exclusively at temporal arteries, 4 had only axillary artery halos, 5 had both districts involved. Temporal artery abnormalities at the time of referral was a predictor of positive CDS (43.8 vs. 10.0%; *P* = 0.046), and it was strongly correlated to a positive halo sign at the level of the temporal arteries (47.7 vs. 7.1%; *P* = 0.006), as well as to bilateral halo sign (51.7 vs. 24.1%; *P* = 0.03).

Forty-two patients who underwent CDS (66.7%) had previously received GC for a median time of 12 days (IQR 3–101). Twenty-two patients (34.9%) had been treated with high-dose GC therapy (≥30 mg/day) for a median time of 4 days (IQR 3–8). The median prednisone-equivalent dosage on the day of scan was 37.5 mg/day (IQR 10–50).

CDS sensitivity was lower in patients with high-dose GC doses protracted for >5 days (*N* = 10), reducing to 60.0% (95% C.I. 26.2–87.8%).

### Follow-Up and Relapses

The median follow-up duration was 3.2 years (IQR 1.0–5.9 years), shorter in the FTA group compared with the conventional one [0.9 years (IQR 0.2–2.0) vs. 5.0 years (IQR 3.5–8.7); *P* < 0.001].

During the follow-up, 82 patients relapsed (51.3%). The median time to disease reoccurrence was 8 months (IQR 3–20 months). The calculated relapse rate per 10 person-years was 3.0 (95% C.I. 2.6–3.4 years). Fifty (31.3%) patients experienced more than one relapse. In the conventional group there were 64 (66%) relapses overall, over a longer follow-up duration; in the FTA 18 (28.6%) relapses were observed. The calculated relapse rate per 10 person-years did not differ between the two cohorts: [2.9 (95% C.I. 2.5–3.4) in the conventional group vs. 3.6 (95% C.I. 2.3–5.2) in the FTA (Table 3).

**Table 3:** Relapse characteristics between the conventional (No FTA) and the FTA cohorts.

	**No FTA (*N* = 64)**	**FTA (*N* = 18)**	***P*-value**
Age at relapse, mean (S.D.), years	70.3 (8.1)	72.7 (7.7)	0.3
Females, *n* (%)	50 (80.0)	12 (66.7)	0.2
Previous PMR, *n* (%)	12 (18.8)	5 (27.7)	0.4
Time to 1st relapse (if any), median (IQR), months	8.5 (3.8–20.3)	7.5 (3.3–10.8)	0.4
Relapse rate per 10 person-years, median (95% C.I.)	2.9 (2.5–3.4)	3.6 (2.3–5.2)	0.3
Any cranial symptom, *n* (%)	41 (64.1)	4 (22.2)	0.002
Any ocular symptom, *n* (%)	4 (6.3)	0 (0.0)	0.3
Permanent visual loss, *n* (%)	0 (0.0)	0 (0.0)	nd
Amaurosis fugax, *n* (%)	4 (6.3)	0 (0.0)	0.3
Jaw claudication, *n* (%)	4 (6.3)	1 (5.6)	0.9
Tongue claudication, *n* (%)	0 (0.0)	0 (0.0)	nd
Scalp tenderness, *n* (%)	3 (4.7)	0 (0.0)	0.4
Headache, *n* (%)	31 (48.4)	3 (16.7)	0.02
PMR, *n* (%)	15 (23.4)	9 (50.0)	0.03
Increased ESR/CRP, *n* (%)	41 (67.2)	12 (66.6)	0.9
ESR, median (IQR), mm/h	36 (25–53)	32 (22–47)	0.2
CRP, median (IQR), mg/L	11.0 (5.4–18.6)	12.5 (5.2–26)	0.9
GC dose, median (IQR), mg/day	12.5 (7.5–25)	13.75 (10–25)	0.4
Immunosuppressive drug, *n* (%)	9 (14.1)	0 (0.0)	0.09
Cranial GCA	48 (75.0)	16 (88.9)	0.2
LV-GCA	16 (25.0)	2 (11.1)	0.2
Ischaemic GCA	17 (26.6)	0 (0.0)	0.01

**Figure 3:**
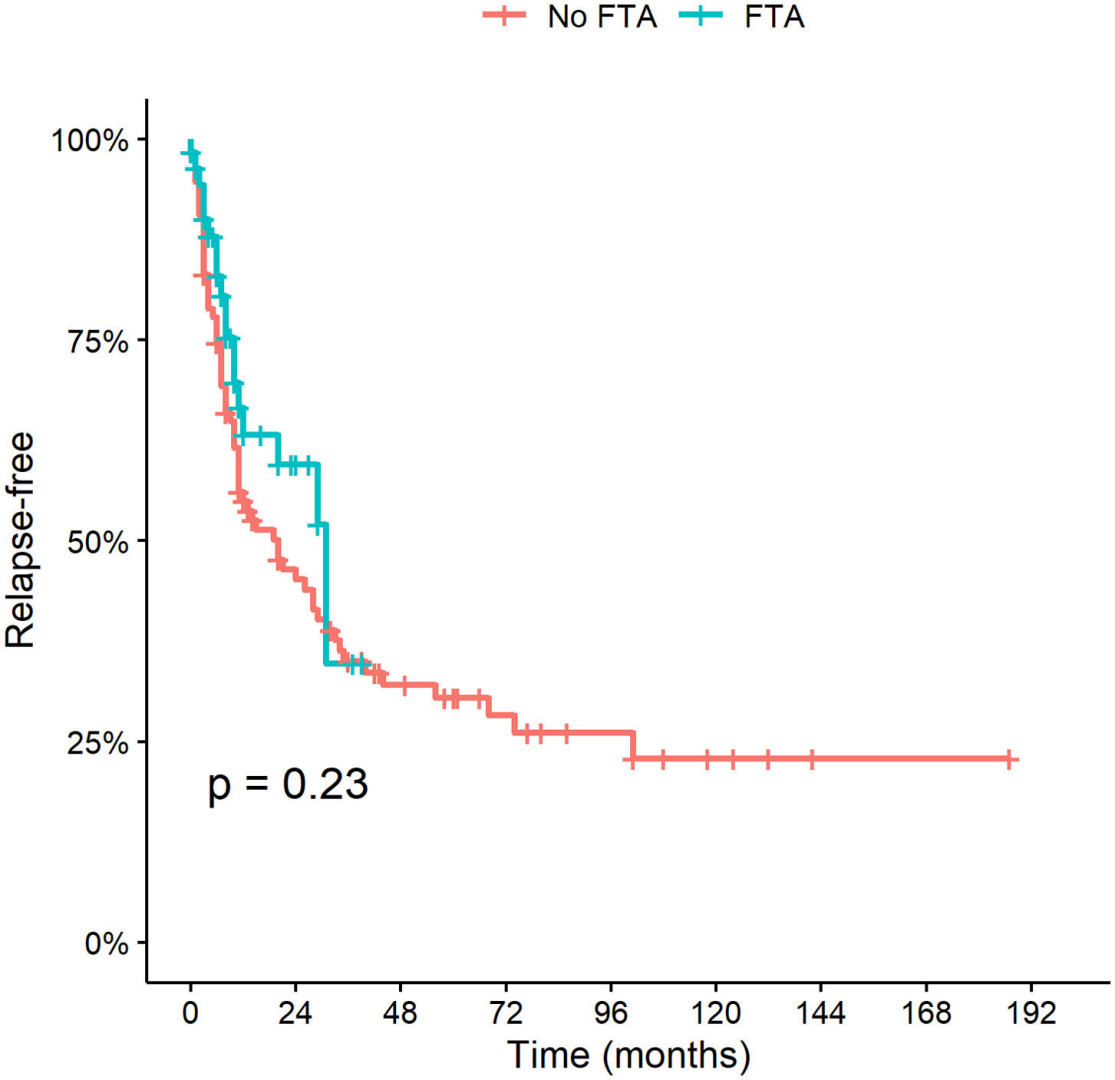
Time to first relapse before and after FTA introduction.

The cumulative incidence of relapse and time to first relapse did not change after FTA was implemented (*P* = 0.23) (Figure 3).

During follow-up there were no new cases of PVL related to disease relapses. Symptoms at the time of first relapse were different between the two groups. In the FTA cohort more patients were presenting with PMR-like symptoms (50.0 vs. 23.4%; *P* = 0.03), whereas cranial symptoms (22.2 vs. 64.1%; *P* = 0.002), especially headache (16.7 vs. 48.4%; *P* = 0.02), were more prominent in the historical cohort. Interestingly, none of the FTA patients with ischaemic GCA relapsed during follow-up (0.0% with ischaemic disease vs. 26.6% without ischaemic GCA; *P* = 0.01).

The ongoing therapy at the time of relapse was similar between the two groups. The median dose of prednisone was 12.5 vs. 13.75 mg/day between the two cohorts (*P* = 0.4).

Out of 18 patients of the FTA group with a confirmed GCA relapse, 14 CDS scans were performed (77.8%). Six scans had signs of active disease (42.9%) in patients with confirmed relapses. Bilateral halo was found in 2 patients with relapse (33.3%).

Regarding the non-FTA group, 13 patients were evaluated with CDS for relapse assessment. The total number of CDS was 22, with 10 of them showing a halo (45.5%) of which four were bilateral halos (40.0%).

Anemia at the time of diagnosis (Hb <11 g/dL) was associated with a higher relapse risk (HR 1.96 95% C.I. 1.04–3.69; *P* = 0.04). Microcytosis (MCV <80 fL) at the time of diagnosis was found to be a good predictor of early disease relapse (HR 2.80 95% C.I. 2.33–3.26; *P* = 0.03). A platelet count above 450,000/mm^3^ was another predictor of relapse (HR 2.67 95% C.I. 1.24–5.7; *P* = 0.01).

The risk of multiple disease relapses was associated to LV-GCA (HR 2.02 95% C.I. 1.07–3.81; *P* = 0.03).

At the end of the follow-up, 38 patients (23.7%) were taking an adjunctive immunosuppressive (IS) agent (34 methotrexate, 3 Tocilizumab, 1 azathioprine) initiated after a median time of 10 months (IQR 5-29 months). There were no significant differences in the requirements for adjunctive IS between the two cohorts. LV-GCA patients were at higher risk of requiring IS (HR 2.42 95% C.I. 1.16–5.04; *P* = 0.02).

### Chronic Complications

**Figure 4:**
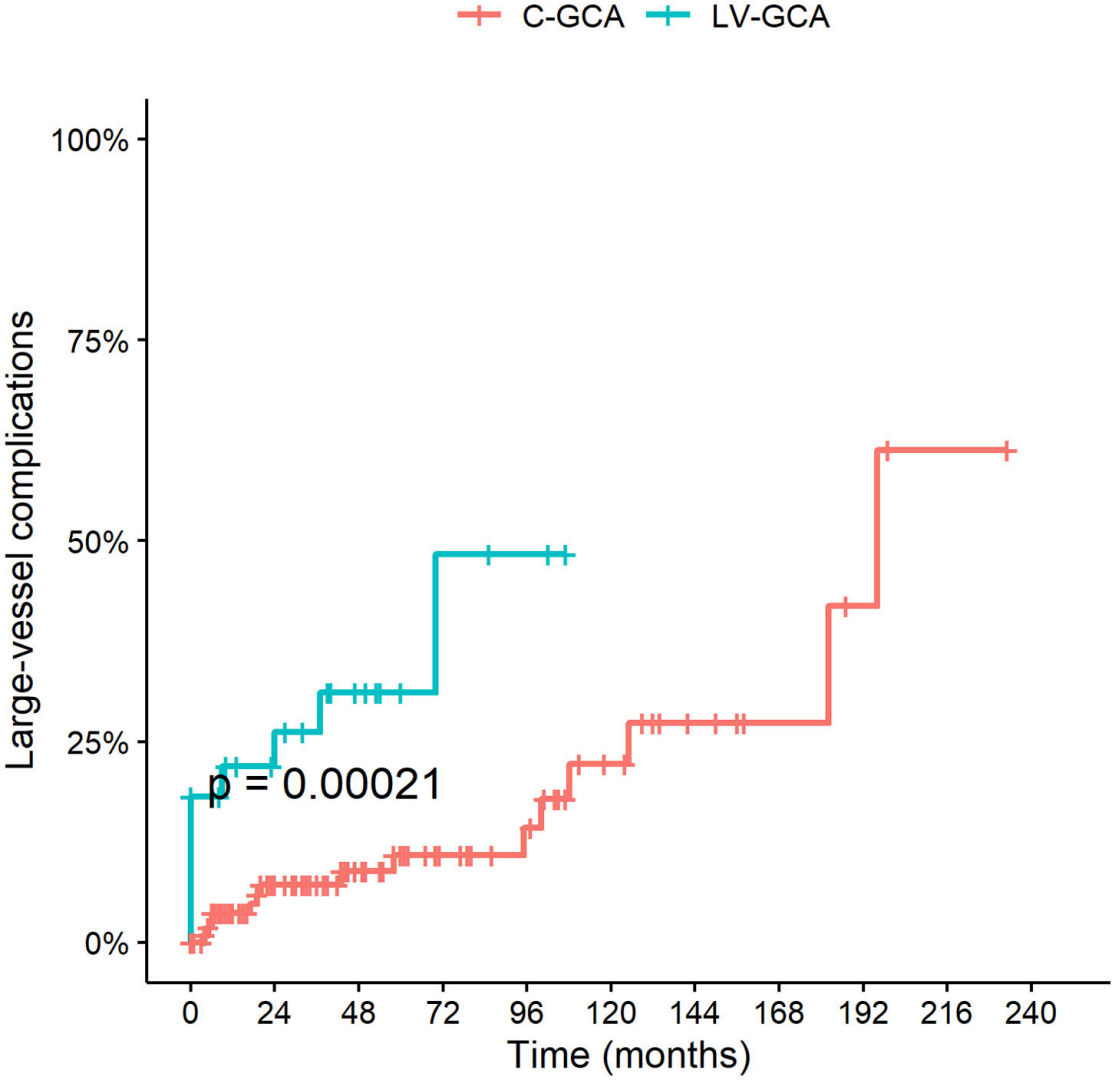
Cumulative incidence of LV complications among LV-GCA compared to C-GCA. LV complications include thoracic/abdominal aortic aneurysm or dissection and stenosis, aneurysm or ectasia of other large arteries.

Large vessel complications (aneurysm, stenosis, vessel ectasia or dissection) were found to be more frequent in patients with LV-GCA (HR 4.58 95% C.I. 1.92–10.92; *P* = 0.0006) (Figure 4). There were no differences between the FTA and the conventional cohort.

## Fast-Track Clinic at the Time of COVID-19

**Figure 5:**
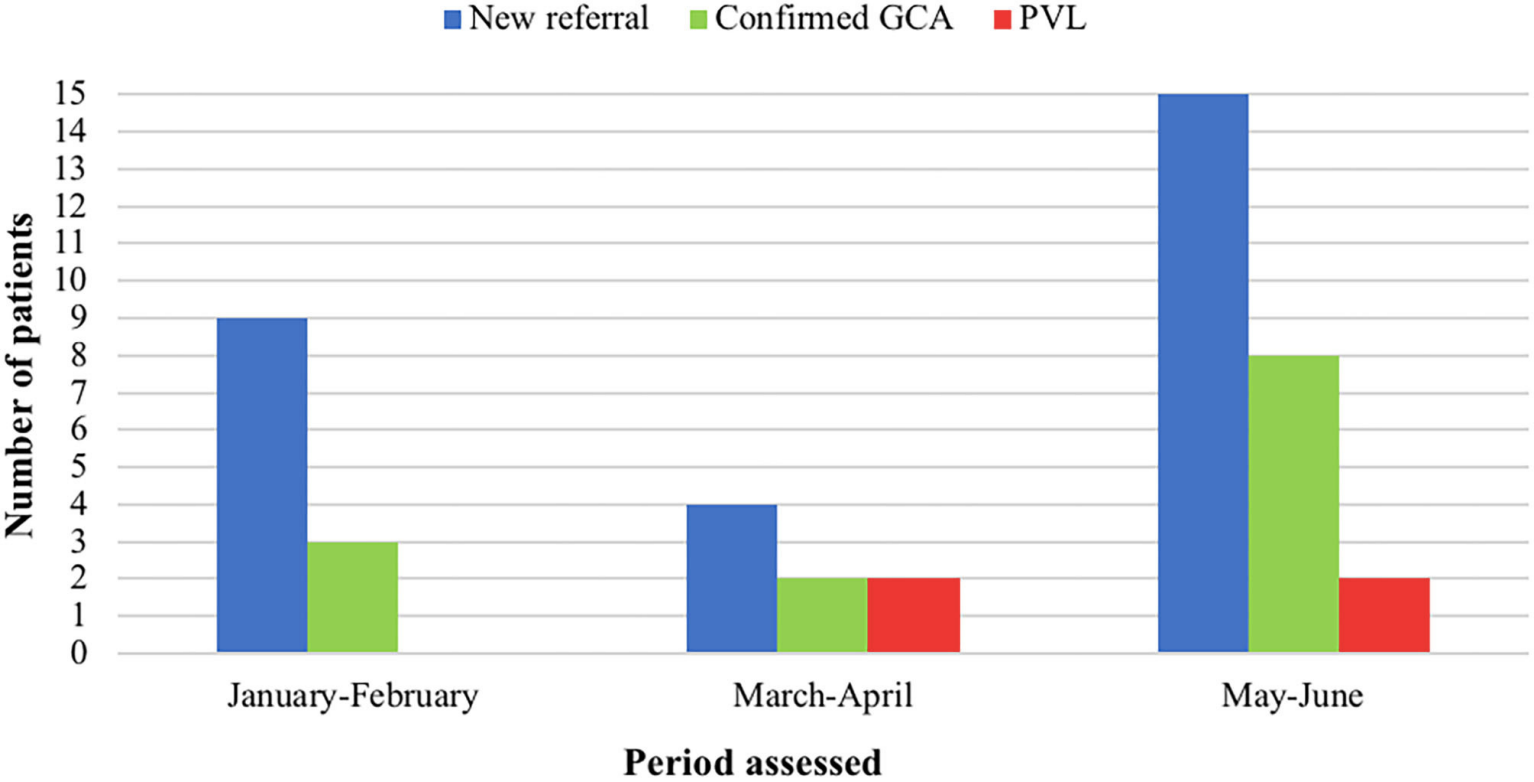
Comparison of the fast-track clinic activity amongst different periods of 2020, including the lockdown months due to COVID-19 pandemic (March-April). GCA, giant cell arteritis; PVL, permanent visual loss.

During the lockdown period due the SARS-CoV-2 pandemic outbreak in Italy (March-April 2020) there was a significant decrease in the number of patients referred to the clinic compared to the preceding months (9 visits compared to 4) and to the corresponding period in the previous year (16 visits compared to 4) despite a regularly operating service (Figure 5). Since the end of the lockdown period the number of new referrals has increased again (15 new visits in the period May-June 2020), nevertheless, a delay in referral has been recorded since the pandemic outbreak. Overall there have been 10 confirmed diagnoses of GCA during this period, female (80%), mean age 76 ± 5 years. The rate of PVL due to GCA has significantly increased during the pandemic, and especially in the rate of bilateral AION which occurred in two patients during the period March-April 2020 compared to one case over the previous 4 years (October 2016-February 2020) of the fast-track clinic activity. PVL occurred in 4 (40%) of GCA patients assessed since March 2020 (vs. 12.7% in the previous FTA period; *P* = 0.03). The duration of symptoms prior to diagnosis since the COVID-19 outbreak in patients developing PVL has increased to 23 days (IQR 15–56) compared to 7 days (IQR 4–10) of the normal FTA activity; the two patients developing bilateral blindness were referred after a mean of 31 days since the symptoms onset.

## Discussion

Our data confirm that FTA including CDS assessment is an innovative disease-modifying strategy leading to a significant reduction of PVL and related disability. The effectiveness of the FTA approach has been further highlighted in our cohort by the significant increase in diagnostic delay and occurrence of PVL, including bilateral blindness, observed during the COVID-19 pandemic due to the patients', and possibly care givers' fear of seeking medical attention and attending the hospital. The use of CDS for the diagnosis of GCA has been first described in 1997 (12), however only in recent years there has been a formal validation and consensus of the role of ultrasound in the management of LVV (9, 13). The recognition of CDS as the first imaging modality in patients with cranial GCA was only included in International recommendations 2 years ago (9, 13). In Centers with the adequate expertise and machine equipment and settings, ultrasound has replaced TAB for the diagnosis of patients with suspected GCA and a highly suggestive clinical picture (7, 13, 14) As demonstrated by the change of practice in our two cohorts, the need for TAB reduced by 93% since the use of CDS as part of the FTA clinic. CDS has the advantage of being a quick, repeatable, low-cost procedure that allows to assess the whole length of the temporal artery (reducing false negatives related to the skipped nature of GCA inflammation) and to extent the examination to other cranial or extra-cranial arteries, optimizing the diagnostic yield (15, 16). CDS has been demonstrated to be more sensitive compared to TAB in the diagnosis of GCA (14), as confirmed by our study. The implementation of CDS into FTA clinics allowing for a prompt and direct assessment and interpretation of imaging findings by the treating physician has led to the demonstration that very early diagnosis with the aid of CDS can significantly reduce the risk of PVL in patients with GCA (6, 17). Our data highlight the importance of early diagnostic assessment and treatment initiation through FTA, but also of early referral through the prompt recognition of prodrome symptoms possibly preceding the occurrence of ischaemic complications of GCA. In our cohort, patients assessed with FTA had less frequent comorbidities albeit being generally older. This characteristic might have contributed to reduce the confounding effect in case of presentation with unspecific symptoms and could have led to a more rapid referral compared to the conventional practice group. Blindness has been described in 15–30% of patients with GCA and is a complication that mostly occurs at the early stages of the disease, often being the presenting symptom leading to the diagnosis (18). Nevertheless, up to 28% of patients with PVL report premonitory reversible transient visual symptoms that can prompt the urgent referral if properly investigated and recognized (18). Other factors associated with PVL include jaw claudication, absence of constitutional symptoms or milder elevation of inflammatory markers (19, 20). Visual loss is usually irreversible in GCA, however, early initiation of GC, particularly if initiated within the 1st day of visual symptoms has been reported as the sole prognostic factor for a partial improvement of visual loss (9, 19). Diagnostic delay is known to be associated with the occurrence of bilateral PVL as confirmed by our data observed during COVID-19 outbreak (19). Clinical suspicion avoiding over-reliance on temporal headache alone is key to the early recognition of GCA. In our FTA cohort some symptoms were recorded as more frequent such as tongue claudication, scalp tenderness or weight loss. It is possible that patients with a clinical presentation more easily resembling GCA would be referred more rapidly, however, increased attention to rarer or less specific symptoms of GCA is pivotal to optimize the clinical suspicion. To further improve the outcome of LVV, the expedited process allowing for early access to specialist evaluation and confirmatory investigational tests obtained with FTA should be paralleled by educational programmes and clear recommendations for fast-track referral offered to primary care and other relevant specialists to further reduced the symptom latency period (21, 22). An improvement of the general public awareness of GCA could also be beneficial to reduce the diagnostic delay. During the COVID-19 pandemic several medical emergencies, including acute coronary syndromes, have recorded an unusual decrease in hospitalization and increased out-of-hospital mortality as a result of avoidance or delay in seeking medical attention (23, 24). Similarly, we have observed a significant increase in the rate of ischaemic complications (with 40% of PVL), including bilateral blindness with two new cases over a period of 2 months, compared to only one case over the 4-year activity of the FTA (25). The duration of symptom prior to the first evaluation for suspected GCA has increased during the COVID-19 outbreak despite a regularly operating fast-track clinic. The negative outcome of newly diagnosed patients with GCA observed during SARS-CoV-2 outbreak confirms once more that urgent referral and FTA are key in the management of LVV with a significant change in visual prognosis.

Nonetheless, our data suggest that FTA does not impact the long-term outcomes and relapse-risk. Our findings are in line with previous reports suggesting that early diagnosis of GCA does not seem to significantly improve the future risk of relapse (26). Predictors of relapse in LVV are poorly understood. Our data confirm that markers of inflammation, baseline anemia, and LV-GCA are associated with a higher risk of relapse (27–30). Increasing interest is emerging to find different predictors of relapse or future disease complications as part of the FTA approach, including quantitative analysis of CDS findings suggestive of more extensive or more severe vascular involvement at disease onset (31). A recently proposed ultrasonographic score combining information on the number of sites with halo and the halo thickness was suggested to be associated with ocular ischaemia (32). Nevertheless, to date, no reliable imaging biomarker has been identified to predict the risk of relapse or future ischaemic complications during follow-up (33, 34).

Our study has some limitations, including the retrospective collection of some of the data. Moreover, comparisons between the two cohorts regarding some of the rarer complications of GCA might have been limited by the small sample size.

The unmodified disease course despite the FTA and early diagnosis strategies in GCA suggest that a change in therapeutic strategy should be applied since the early stages of the disease to significantly modify the long-term outcome. Available evidence demonstrated a glucocorticoid-sparing effect and efficacy in reducing the risk of relapse in newly diagnosed or relapsing patients with GCA treated with methotrexate or tocilizumab (35, 36). Nevertheless, in clinical practice patient-tailored evaluations of safety and cost-effectiveness issues are often taken into consideration in this elderly population group treated with concomitant high-dose glucocorticoids. Current recommendations suggest to reserve adjunctive therapy to selected patients with refractory or relapsing disease or with the presence or increased risk of glucocorticoid related adverse events and complications. A risk stratification process to select patients with poor prognosis to be treated more intensively upfront still requires further research. Data obtained from the FTA clinics suggest that the window of opportunity to obtain long-term modifications of the disease process in GCA is exceedingly short and should probably be associated with an optimization of the disease awareness to further accelerate referral and with a tailored therapeutic approach to potentiate treatment in patients at higher risk of relapse and complications.

## Conclusion

FTA including CDS evaluation contributed to a substantial reduction of the PVL risk in GCA by shortening the time to diagnosis and treatment initiation. Relapse rate and LV-complications did not change upon FTA introduction, highlighting the need for better disease activity monitoring strategies and risk stratification at disease onset that would predict the occurrence of relapse during glucocorticoid de-escalation.

## Data Availability Statement

The raw data supporting the conclusions of this article will be made available by the authors, without undue reservation.

## Ethics Statement

The studies involving human participants were reviewed and approved by University of Pavia Ethical Committee. The patients/participants provided their written informed consent to participate in this study.

## Author Contributions

SM, AB, EB, PD, and CM contributed equally to the ideation and realization of the research project and critically reviewed the manuscript. SM and AB wrote the manuscript. All authors contributed to the article and approved the submitted version.

## Conflict of Interest

The authors declare that the research was conducted in the absence of any commercial or financial relationships that could be construed as a potential conflict of interest.

## Supplementary Material

Supplementary Figure 1 Cumulative risk of blindness according to symptom latency in the whole cohort of patients (convenational approach, fast-track, and fast-track during COVID-19 pandemic).

Supplementary Figure 2 Kaplan-Meyer curves of visual survival in the conventional group compared to the fast-track approach.
