# Supplementary figures and images for: Fast-Track Ultrasound Clinic for the Diagnosis of Giant Cell Arteritis Changes the Prognosis of the Disease but Not the Risk of Future Relapse
Source: Front Med (Lausanne). 2020 Dec 8;7:589794. doi: 10.3389/fmed.2020.589794 (PMC7753207; doi:10.3389/fmed.2020.589794)

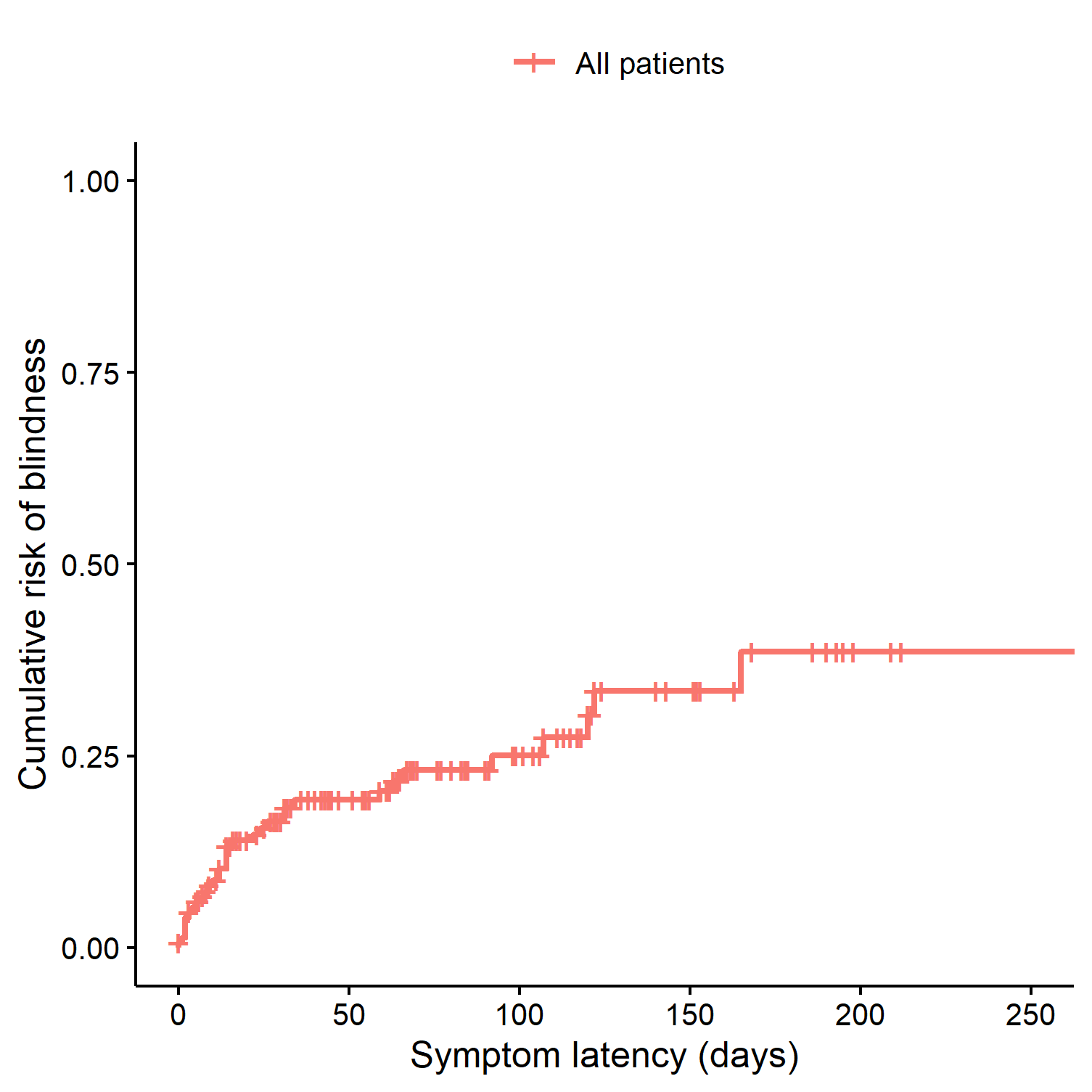

Supplement: Supplementary Figure 1 — Cumulative risk of blindness according to symptom latency in the whole cohort of patients (convenational approach, fast-track, and fast-track during COVID-19 pandemic). [file Image_1.TIFF]

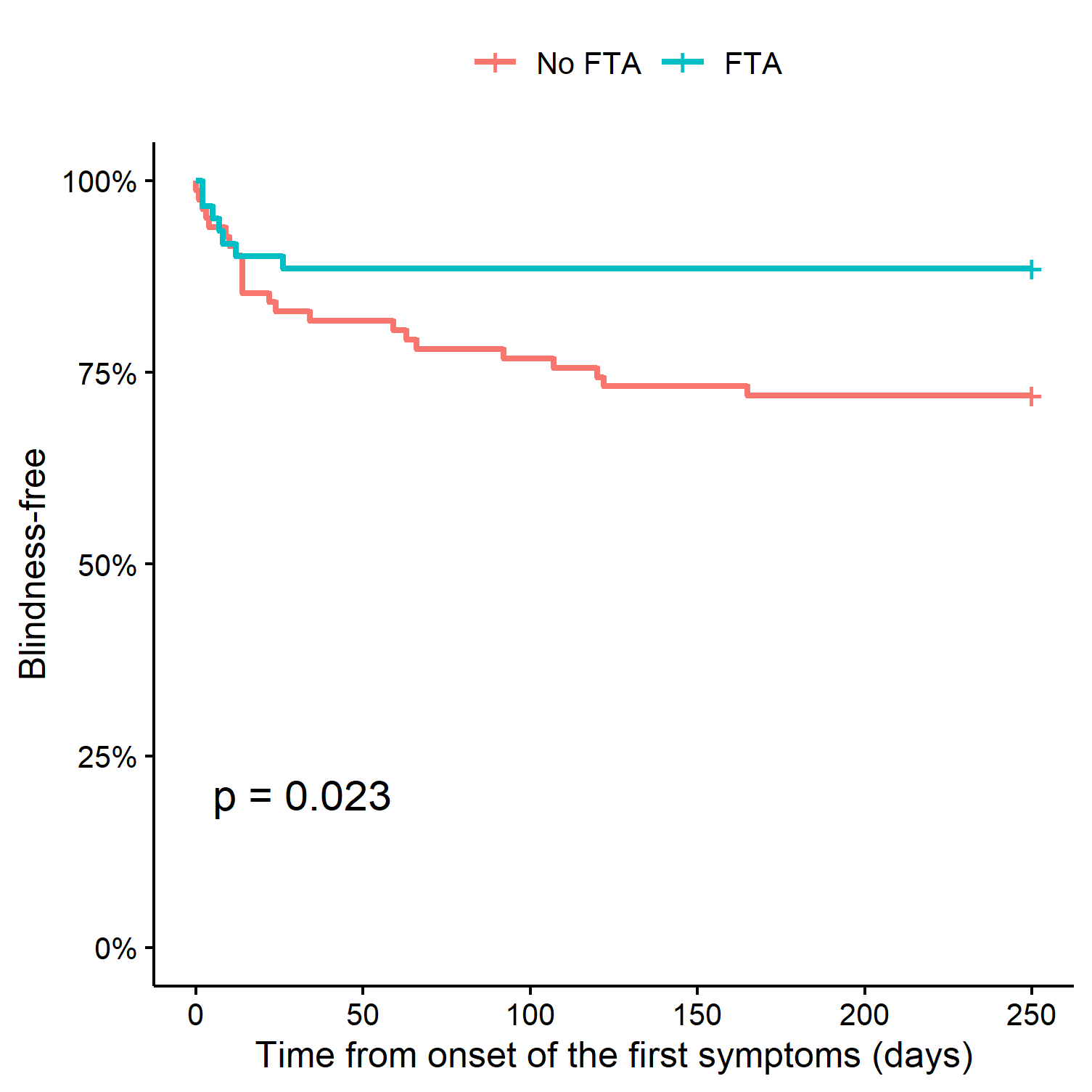

Supplement: Supplementary Figure 2 — Kaplan-Meyer curves of visual survival in the conventional group compared to the fast-track approach. [file Image_2.TIFF]
